# Wearable Glove with Enhanced Sensitivity Based on Push–Pull Optical Fiber Sensor

**DOI:** 10.3390/bios15070414

**Published:** 2025-06-27

**Authors:** Qi Xia, Xiaotong Zhang, Hongye Wang, Libo Yuan, Tingting Yuan

**Affiliations:** 1 Key Laboratory of In-Fiber Integrated Optics of Ministry of Education, College of Physics and Optoelectronic Engineering, Harbin Engineering University, Harbin 150001, China; xiaqi_hrb@163.com (Q.X.);; 2 College Health Science and Environmental Engineering, Shenzhen Technology University, Shenzhen 518118, China; 3 School of Electronic and Information Engineering, Ningbo University of Technology, Ningbo 315211, China; 4 Photonics Research Center, School of Optoelectronic Engineering, Guilin University of Electronic Technology, Guilin 541004, China; 5 Future Technology School, Shenzhen Technology University, Shenzhen 518118, China

**Keywords:** wearable biosensors, wearable glove, multi-core fiber, fiber Bragg grating, bending sensor

## Abstract

Hand motion monitoring plays a vital role in medical rehabilitation, sports training, and human–computer interaction. High-sensitivity wearable biosensors are essential for accurate gesture recognition and precise motion analysis. In this work, we propose a high-sensitivity wearable glove based on a push–pull optical fiber sensor, designed to enhance the sensitivity and accuracy of hand motion biosensing. The sensor employs diagonal core reflectors fabricated at the tip of a four-core fiber, which interconnect symmetric fiber channels to form a push–pull sensing mechanism. This mechanism induces opposite wavelength shifts in fiber Bragg gratings positioned symmetrically under bending, effectively decoupling temperature and strain effects while significantly enhancing bending sensitivity. Experimental results demonstrate superior bending-sensing performance, establishing a solid foundation for high-precision gesture recognition. The integrated wearable glove offers a compact, flexible structure and straightforward fabrication process, with promising applications in precision medicine, intelligent human–machine interaction, virtual reality, and continuous health monitoring.

## 1. Introduction

The human hand, located at the distal end of the upper limb, is one of the most flexible and frequently used organs of the body. Together with the wrist, the hand provides both dexterity and essential support, playing a vital role in daily life, manual labor, and fine motor tasks [1]. The hand not only performs a variety of functions, such as grasping, manipulating, and expressing emotions, but also plays a critical role in the exchange of information between the nervous system and the external environment [2]. Due to its complex structure and refined functionality, the health condition of the hand often directly affects an individual’s quality of life and work capacity [3]. Patients with impaired hand function are typically treated with physical therapy, where exercises such as gripping or pinching help to restore and improve the motor abilities of the hand [4,5].

The assessment of rehabilitation status is often complex, prompting the development of various solutions, including machine vision-based approaches [6], inertial measurement unit (IMU) [7], and wearable device-based approaches [8,9]. Byberi et al. developed a wearable device named “GloveSense”, which was fabricated by integrating conductive wires into a regular glove [10]. Wong et al. proposed a prototype of a wearable capacitive sensor unit that acquires capacitance values from electrodes placed on the phalanges. Each gesture corresponds to a specific capacitance value, and two machine learning algorithms were employed for gesture recognition [11]. Among wearable devices, optical fiber sensors have garnered significant attention due to their compact size, ease of integration, high stability, excellent sensitivity, immunity to electromagnetic interference, and compatibility with wavelength-division multiplexing [12,13,14,15,16]. These characteristics make them particularly well-suited for use in wearable gesture-sensing gloves, offering advantages such as compactness, flexibility, and lightweight design [17]. In human–computer interaction (HCI) scenarios, optical fiber sensors have already demonstrated outstanding performance [18,19,20].

In the field of wearable glove research based on optical fiber Bragg grating (FBG) technology, numerous scholars have made significant progress and achieved notable results [21,22,23,24,25,26]. Guo et al. proposed a method for detecting finger movements using FBG embedded in polyimide substrates [27]. Xiong et al. developed a technique in which FBGs are embedded in polyimide (PI) films and aligned along the normal direction of muscle surfaces to recognize gestures by detecting muscle activity [28]. Li et al. reported a method of embedding fiber Bragg gratings (FBGs) in silicone tubes for joint motion monitoring. By integrating sensors into fingers, arms, and knees, and combining them with wave-shaped protective bands, the system can monitor joint bending and distinguish different joint motions [29]. Yi et al. proposed a technique for detecting wrist joint posture using FBG sensors. The FBG, integrated with a composite substrate of polydimethylsiloxane (PDMS) and PI, is fixed on the skin above specific forearm muscles to monitor muscle deformation caused by wrist movement [23]. Xiao et al. designed an FBG sensor embedded in PDMS silicone elastomer to detect motions such as wrist tilting, finger bending, and mouth movements [25].

In the field of hand motion detection, data gloves, wristbands, and similar devices offer real-time monitoring capabilities. However, their limited sensitivity and complex structures can hinder the natural flexibility of hand movements. To address these challenges, we propose a wearable glove based on shape reconstruction using multi-core fiber Bragg gratings (MCF-FBGs), which are embedded in polyimide-coated quartz tubes for hand motion sensing. The core structure utilizes MCF-FBGs as vector bending sensors, featuring high integration, simple and flexible deployment, and excellent stability.

In earlier studies, various vector bending sensors based on single-core optical fibers were developed, such as FBG [30,31], long-period gratings [32,33], and fiber interferometers [34,35]. However, due to the structural limitations of single-core fibers, these sensors generally exhibit limited directional discrimination and can only detect uniaxial bending—either in the forward or reverse direction. In recent years, vector bending sensors based on multi-core fiber (MCF) have developed rapidly, offering superior integration, compactness, and mechanical strength compared to single-core fiber sensors [36,37].

For a single sensing location, compared to a conventional single-core fiber sensor, the wearable glove based on MCF shape reconstruction requires at least four demodulation channels to achieve full spatial shape recovery. To address this limitation, a four-core fiber (FCF) with two orthogonally symmetric core pairs was utilized. Diagonal core reflectors were inscribed at the fiber tip to interconnect the symmetric cores, thereby constructing a push–pull sensing configuration. Through a tip-reflecting coupler (TRC), the number of demodulation channels required by the system was halved, making multi-channel demodulation feasible while also doubling the sensitivity. The push–pull sensing configuration improves sensitivity by exploiting the opposite wavelength shifts exhibited by the FBGs in the symmetric cores under bending deformation [38,39,40]. Experimental results have validated the temperature-decoupling capability and bending-sensing performance of this push–pull optical fiber sensor. The wearable glove based on this sensor offers advantages such as a simple structure, ease of fabrication, and high sensitivity. This represents a promising advancement in wearable technology, with broad application potential in precision medicine and intelligent interaction. Furthermore, it holds significant promise for use in areas such as sports training, human–computer interaction, and virtual reality.

## 2. Principles and Fabrication of Sensing Systems

### 2.1. Subsection Sensor System Design

**Figure 1:**
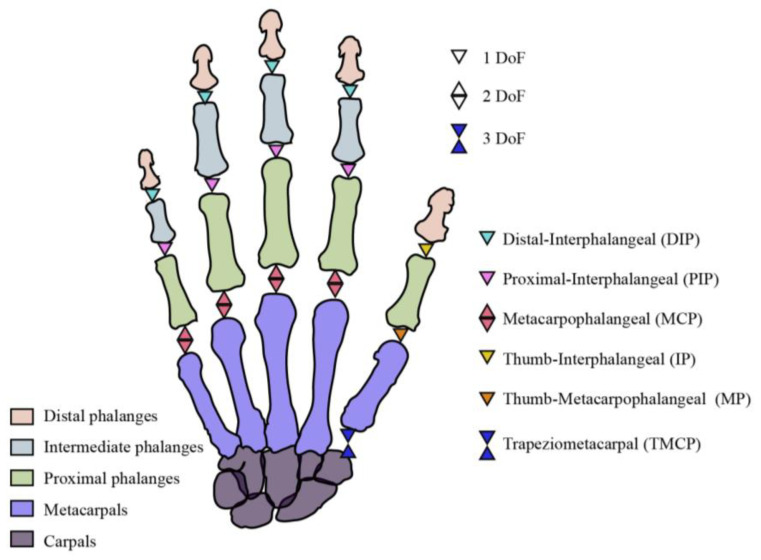
Hand fingers, joints, and related DoFs.

Figure 1 illustrates the anatomical structure of the human hand joints along with their associated degrees of freedom (DoFs), which are commonly used to characterize hand motion. Each finger’s distal interphalangeal (DIP) and proximal interphalangeal (PIP) joints possess one DoF, enabling flexion and extension. The metacarpophalangeal (MCP) joints have two DoFs, allowing for both flexion/extension and abduction/adduction. Additionally, the trapeziometacarpal (TMCP) joint of the thumb provides a third DoF, permitting longitudinal rotation as the thumb moves into opposition with the fingers [41]. The presence of these multi-DoF joints highlights the necessity for vectorial bending detection to accurately capture the complex and multi-directional movements of the human hand.

**Figure 2:**
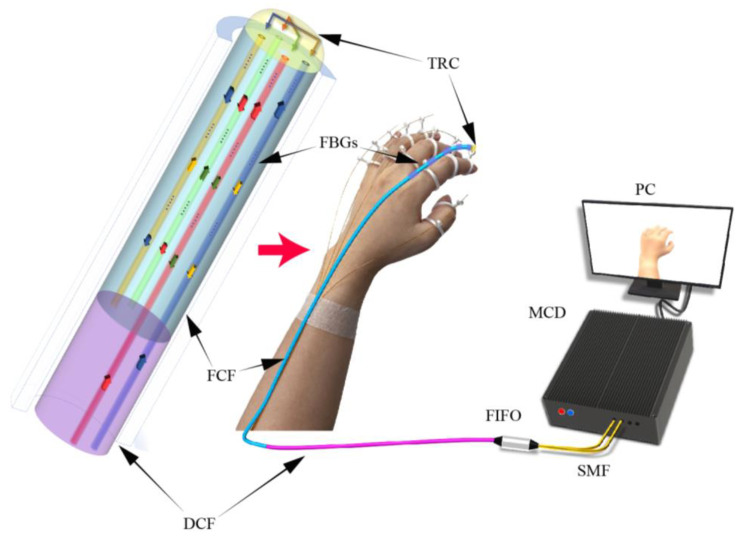
Schematic diagram of a wearable glove system based on push–pull fiber-optic sensing.

In conventional FCF-based wearable glove systems, each single-finger sensor typically requires four independent demodulation channels. As a result, a full glove system demands 20 separate channels, while the sensitivity of each single-channel demodulation remains limited. To overcome these challenges, we designed a wearable gesture-sensing glove based on a push–pull optical fiber sensor, as illustrated in Figure 2. This design not only enhances measurement sensitivity but also reduces the required number of demodulation channels by half. The system comprises an MCF-based wearable glove sensor, fan-in/fan-out (FIFO) couplers, and a multi-channel demodulator (MCD, F210-16, ChangheGuanglian, Shenzhen, China). The MCF glove sensor is constructed using FCF and TRC, and is connected to the MCD via dual-core fiber (DCF) and FIFO.

FBGs are inscribed at identical axial positions in all four cores of the FCF using a defocused phase mask technique. Opposing cores in the FCF are optically connected through two reflective paths established within the TRC, as indicated by the red and blue arrows in Figure 2. As illustrated by the yellow and green arrows, the reflected signals from the FBGs in cores 3 and 4 are, respectively, coupled into cores 1 and 2. Each channel of the MCD receives and demodulates the reflected signals from a pair of FBGs located in two optically interconnected fiber cores.

### 2.2. Principle of the FBG Sensing

The reflected wavelength of FBG $\lambda_{B}$ changes with the period of the grating Λ and the effective refractive index of the core mode $n_{eff}$. The wavelength shift in the FBG $\Delta \lambda_{B}$ can be expressed as follows:

(1) $$\Delta \lambda_{B}=2\Lambda \Delta n_{eff}+2n_{eff}\Delta \Lambda$$

The period change in FBG and the elasto-optical effect make the FBG reflected wavelength sensitive to strain ε and pressure ΔP variations. Temperature ΔT affects the reflected wavelength of FBG due to thermal expansion and thermo-optic effects.

(2) $$\left\{\begin{array}{l} \Delta \lambda_{BT}=K_{T}\cdot \Delta T\\ \Delta \lambda_{B\varepsilon }=K_{\varepsilon }\cdot \varepsilon \\ \Delta \lambda_{BP}=K_{P}\cdot \Delta P \end{array}\right.$$

where $K_{T}$, $K_{\varepsilon }$, and $K_{P}$, respectively, represent the temperature sensitivity, strain sensitivity, and pressure sensitivity of FBG. $\Delta \lambda_{BT}$, $\Delta \lambda_{B\varepsilon }$, and $\Delta \lambda_{BP}$, respectively, represent the wavelength shifts caused by temperature, strain, and pressure.

**Figure 3:**
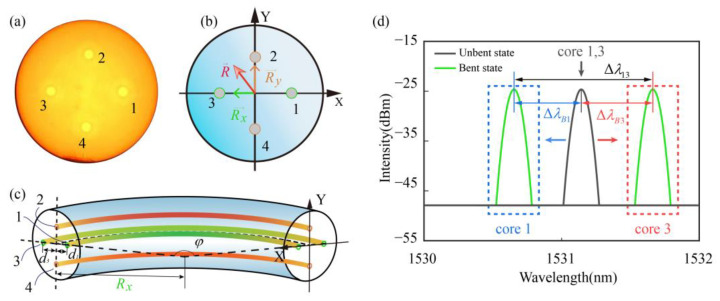
Schematic illustration of multi-core fiber bending sensing: (**a**) Microscopic image of the FCF cross-section. (**b**) Schematic diagram of the FCF cross-section under bending. (**c**) Schematic diagram of Bragg gratings in the FCF under bending. (**d**) Schematic diagram of the Bragg grating spectrum of cores 1 and 3 in the FCF under bending.

For the FCF shown in Figure 3a, the core diameter is approximately 8.5 μm, and the cladding diameter is approximately 125 μm. The distance between core 1, core 3, and core 2, core 4 is approximately 65 μm. Take the direction of fiber core 3 pointing to fiber core 1 as the X-axis positive direction. Take the direction of fiber core 4 pointing to fiber core 2 as the Y-axis positive direction, and establish a right-angle coordinate system for the curved cross-section as shown in the relative Figure 3b. The wavelength shift in FBGs on different fiber cores is

(3) $$\Delta \lambda_{Bi}=K_{\varepsilon i}\varepsilon_{i}+K_{Ti}\Delta T_{i}+K_{Pi}\cdot \Delta P_{i}(i=1,2,3,4)$$

Strain on the fiber core $\varepsilon_{i}$ can be divided into strain generated by the bending of the fiber core $\varepsilon_{ri}$ and strain generated by the axial tension and compression of the fiber core $\varepsilon_{zi}$, i.e., $\varepsilon_{i}=\varepsilon_{ri}+\varepsilon_{zi}$. FBGs at the same position on the FCF have the same $\varepsilon_{zi}$, $\Delta T_{i}$, and $\Delta P_{i}$. $K_{\varepsilon i}$, $K_{Ti}$, and $K_{Pi}$ can be calibrated through experimentation.

Cores 1 and 3 form one sensing channel through the action of the TRC. Similarly, cores 2 and 4 constitute a second channel. This configuration establishes a push–pull sensing system. From Equation (3), we obtain the following:

(4) $$\left\{\begin{array}{l} \Delta \lambda_{13}=\Delta \lambda_{B1}-\Delta \lambda_{B3}\\ =K_{\varepsilon 1}\varepsilon_{r1}-K_{\varepsilon 3}\varepsilon_{r3}+\varepsilon_{z}(K_{\varepsilon 1}-K_{\varepsilon 3})+\Delta T(K_{T1}-K_{T3})\\ \Delta \lambda_{24}=\Delta \lambda_{B2}-\Delta \lambda_{B4}\\ =K_{\varepsilon 2}\varepsilon_{r2}-K_{\varepsilon 4}\varepsilon_{r4}+\varepsilon_{z}(K_{\varepsilon 2}-K_{\varepsilon 4})+\Delta T(K_{T2}-K_{T4}) \end{array}\right.$$

The bending of FBGs in different cores is illustrated in Figure 3c. Taking cores 1 and 3 as an example, the corresponding spectral illustration is shown in Figure 3d. A blue shift $\Delta \lambda_{B1}$ and red shift $\Delta \lambda_{B3}$ occur in the symmetric cores under bending, and for the channel formed by cores 1 and 3, the differential wavelength change $\Delta \lambda_{13}$ is nearly twice that observed in a single core, demonstrating the effectiveness of the push–pull sensing mechanism. Let $d_{i}$ denote the distance from core i to the central axis of symmetry of the fiber, and let $d_{ij}$ represent the distance between cores i and j. The relationship between the bending radius $R_{x}$ along the X-axis and the bending strains $\varepsilon_{r1}$ and $\varepsilon_{r3}$ in cores 1 and 3 is given by the following:

(5) $$\begin{array}{l} K_{\varepsilon 1}\varepsilon_{r1}-K_{\varepsilon 3}\varepsilon_{r3}=\frac{K_{\varepsilon 1}[(R_{x}+d_{1})\varphi -R_{x}\varphi ]}{R_{x}\varphi }-\frac{K_{\varepsilon 3}[(R_{x}-d_{3})\varphi -R_{x}\varphi ]}{R_{x}\varphi }\\ =\frac{K_{\varepsilon 1}d_{1}+K_{\varepsilon 3}d_{3}}{R_{x}} \end{array}$$

(6) $$\left\{\begin{array}{l} R_{x}=\frac{K_{\varepsilon 1}d_{1}+K_{\varepsilon 3}d_{3}}{K_{\varepsilon 1}\varepsilon_{r1}-K_{\varepsilon 3}\varepsilon_{r3}}\\ R_{y}=\frac{K_{\varepsilon 2}d_{2}+K_{\varepsilon 4}d_{4}}{K_{\varepsilon 2}\varepsilon_{r2}-K_{\varepsilon 4}\varepsilon_{r4}} \end{array}\right.$$

When $d_{1}=d_{3}$, it can be seen from Equation (5) that the sensitivity of push–pull bending measurement is doubled compared to single-core measurement. The bending directions of cores 1, 3 and cores 2, 4 can be determined by the positivity and negativity of $\varepsilon_{r1}$ and $\varepsilon_{r3}$. Combined with Equation (6), we can obtain the bending vector $\vec{R_{x}}$ in the X-axis direction and the bending vector $\vec{R_{y}}$ in the Y-axis direction. The bending vector $\vec{R}$ at this position is as follows:

(7) $$\vec{R}=\vec{R_{x}}+\vec{R_{y}}$$

(8) $$\theta =\arctan\left(\frac{\vec{R_{y}}}{\vec{R_{x}}}\right)$$

where θ is the angle between the fiber bending direction and the positive direction of the X-axis.

### 2.3. Fabrication of Sensor

**Figure 4:**
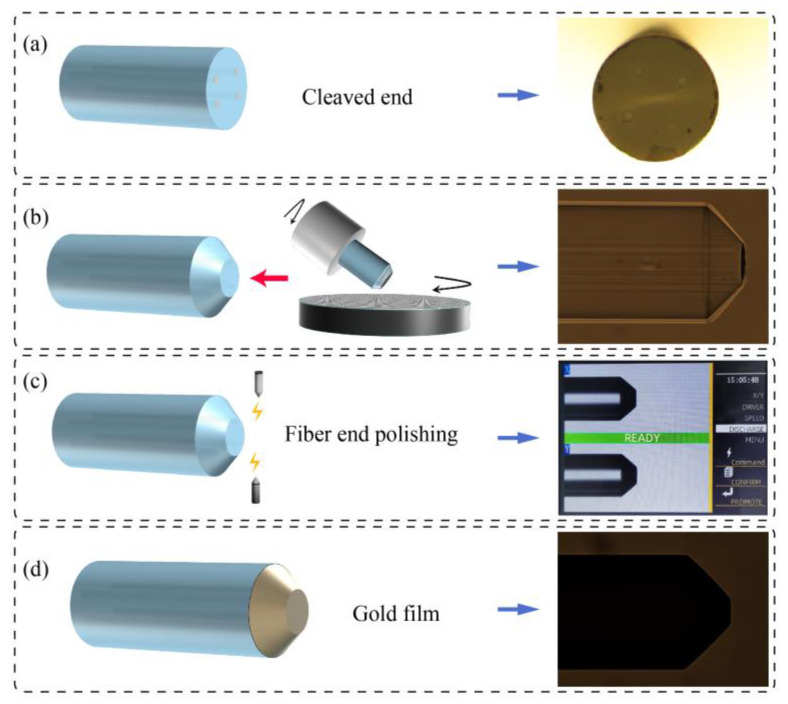
Fabrication of FCF tip reflection coupler: (**a**) Bare fiber cleaving. (**b**) Polishing of optical fiber. (**c**) Optimization of cone. (**d**) Gold-plated film on fiber end.

To enable push–pull optical coupling between the fiber cores, an FCF TRC was fabricated. The fabrication process is illustrated in Figure 4. First, the FCF was cleaved, as shown in Figure 4a. The prepared fiber was mounted on the rotating fixture of a grinding device, with coarse sandpaper attached to the grinding pad. The angle between the FCF and the sandpaper was set to 45°, a value optimized through simulation, with the simulated coupling efficiency exceeding 85%. After the fiber was ground into a conical shape, it was removed and cleaned using an ultrasonic cleaner.

Subsequently, the FCF was re-mounted onto the grinding device, and the coarse sandpaper was replaced with polishing sandpaper. The fiber was then polished at the same 45° angle, as shown in Figure 4b. Next, the polished 45° cone was placed into an electrode holder (Figure 4c), where an electric arc was used to slightly melt the cone tip by adjusting the discharge voltage and duration, resulting in a smooth surface. Finally, the FCF was placed into a magnetron sputtering chamber, where a thin layer of gold was deposited onto the conical surface, as shown in Figure 4d.

Through this process, the fabrication of the TRC was completed, enabling optical coupling between the symmetric cores of the FCF. Batch production tests showed that the coupling loss of the TRC ranged from −2 dB to −3 dB.

**Figure 5:**
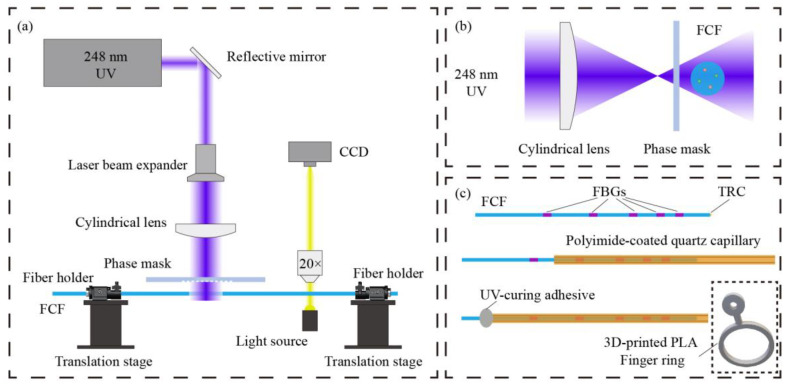
Sensor fabrication process: (**a**) Schematic of FBG inscription using excimer laser. (**b**) Defocused phase mask method. (**c**) Schematic of the wearable glove sensor fabrication process.

We employed ultraviolet laser inscription to fabricate FBGs in the FCF, as illustrated in Figure 5a. The FCF was first placed in a high-pressure hydrogen chamber at 70 °C to enhance its photosensitivity. A KrF excimer laser with a wavelength of 248 nm was then used to simultaneously inscribe FBGs into all four cores. Prior to inscription, the coating at the designated region was removed, and the fiber was mounted on a rotating fiber holder. A CCD camera was used to observe the position of the cores, and the fiber was rotated to avoid shadowing or interference among the cores during inscription. The defocused phase mask method, shown in Figure 5b, was employed for inscription, with careful adjustment of the distance between the fiber and the phase mask. After the FBGs’ inscription, the fiber was recoated to maintain its mechanical integrity. In the experiment, five phase masks with different grating periods were used to inscribe five sets of FBGs in the FCF.

The FCF with inscribed FBGs was inserted into a quartz capillary coated with polyimide. UV-curable adhesive was applied at the junctions between the fiber and the quartz tube to ensure secure fixation. As shown in Figure 5c, we designed a ring-shaped holder made of PLA material, which was fabricated using 3D printing technology. The polyimide-coated quartz capillary was threaded through the ring to complete the assembly of the wearable glove sensor. The polyimide-coated quartz tube offers both excellent straightness and considerable flexibility, making it an ideal material for ensuring continuous shape transmission.

## 3. Sensor Calibration Experiments

### 3.1. Temperature Compensation Characteristic

**Figure 6:**
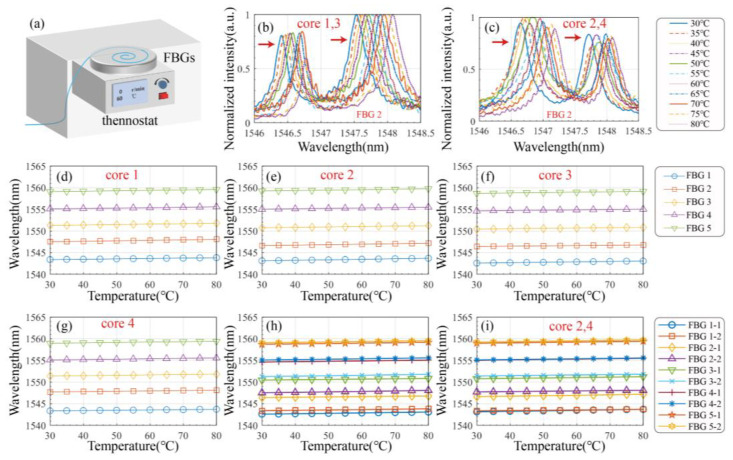
Temperature compensation characteristics, measuring device, and measurement results: (**a**) Schematic of the experimental setup for measuring temperature. Spectrum graph of (**b**) channel 1 and (**c**) channel 2. Relationship between wavelength and temperature for FBGs of fiber (**d**) core 1, (**e**) core 2, (**f**) core 3, (**g**) core 4, (**h**) channel 1, and (**i**) channel 2.

The temperature measurement device is shown in Figure 6a, with the sensor fixed to the thermostat. From Equation (3), we can obtain the following:

(9) $$\left\{\begin{array}{l} \Delta \lambda_{B1}=\Delta \lambda_{B2}=\Delta \lambda_{B3}=\Delta \lambda_{B4}=K_{Ti}\Delta T_{i}(i=1,2,3,4)\\ \Delta \lambda_{13}=\Delta \lambda_{B1}-\Delta \lambda_{B3}\approx 0\\ \Delta \lambda_{24}=\Delta \lambda_{B2}-\Delta \lambda_{B4}\approx 0 \end{array}\right.$$

In Equation (9), since the Bragg wavelengths are nearly identical, the temperature coefficients can be considered approximately equal according to Equations (1) and (2). From Equation (9), it can be observed that with the integration of the TRC, the grating array enables temperature sensing. Compared to traditional sensing approaches, the push–pull configuration facilitates inherent temperature compensation. In the experiment, the thermostat was initialized at 30 °C and incremented by 5 °C at each step until reaching 80 °C. After each temperature increment, a stabilization period of 5 min was allowed before measurements were taken.

Figure 6b,c show the reflection wavelength shifts in FBG2 in channel 1 and channel 2, respectively, as the temperature varied from 30 °C to 80 °C. As illustrated in Figure 6d–g, all the FBGs exhibit a redshift in wavelength with increasing temperature. Linear regression was applied to the data from channels 1 and 2, as shown in Figure 6h,i.

The measured temperature sensitivities of FBG1 to FBG5 over the range of 30 °C to 80 °C were 9.3 pm/°C, 9.1 pm/°C, 8.8 pm/°C, 9.2 pm/°C, and 9.6 pm/°C, respectively, with corresponding R^2^ values of 0.9993, 0.9960, 0.9975, 0.9994, and 0.9992. The primary source of measurement error was the thermal uniformity of the experimental setup. These results demonstrate that the push–pull sensing approach using a multi-core optical fiber effectively enables temperature compensation.

### 3.2. Strain Compensation Characteristic

**Figure 7:**
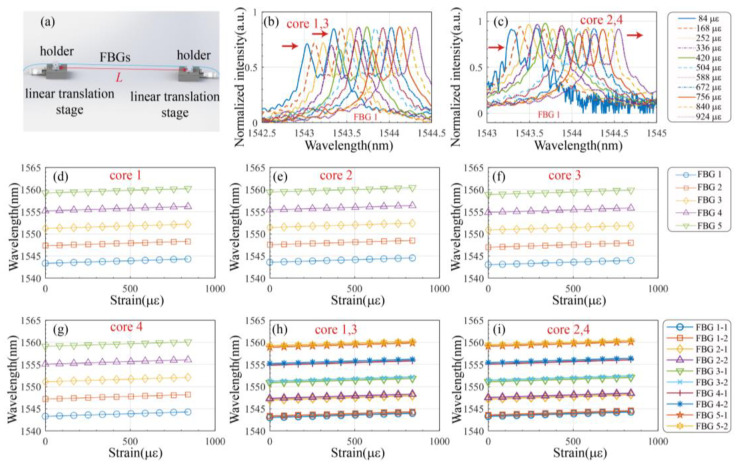
Strain compensation characteristic measuring device and measurement results: (**a**) Schematic of the experimental setup for measuring strain. Spectrum graph of (**b**) channel 1 and (**c**) channel 2. Relationship between wavelength and strain for FBGs of fiber (**d**) core 1, (**e**) core 2, (**f**) core 3, (**g**) core 4, (**h**) channel 1, and (**i**) channel 2.

The strain measurement setup is illustrated in Figure 7a, where the fiber is securely mounted on the displacement stage holder. Based on Equations (2) and (3), the following relationship can be derived.

(10) $$\left\{\begin{array}{l} \Delta \lambda_{B1}=\Delta \lambda_{B2}=\Delta \lambda_{B3}=\Delta \lambda_{B4}=K_{\varepsilon i}\varepsilon_{zi}(i=1,2,3,4)\\ \Delta \lambda_{13}=\Delta \lambda_{B1}-\Delta \lambda_{B3}\approx 0\\ \Delta \lambda_{24}=\Delta \lambda_{B2}-\Delta \lambda_{B4}\approx 0 \end{array}\right.$$

where $\varepsilon_{zi}$ represents the longitudinal strain of the FCF. In Equation (10), similarly, due to the near-identical Bragg wavelengths, the strain coefficients are also approximately equal as derived from Equations (1) and (2).

From Equation (10), it can be observed that the addition of the TRC enables the grating array to measure longitudinal strain, while the push–pull sensing configuration allows for longitudinal strain compensation. Initially, the distance L between the two displacement stages was 1190 mm, and the displacement stage was incrementally moved outward by 0.1 mm each time.

Figure 7b,c display the variation in the FBG1 spectra with increasing strain in channels 1 and 2. Figure 7d through Figure 7g demonstrate that as strain increases, the reflection wavelengths of all the FBGs shift toward longer wavelengths (redshift). Figure 7h,i show the corresponding changes in grating reflection wavelengths with strain in channels 1 and 2. Linear regression analysis of the data points in Figure 7h,i yields the results summarized in Table 1. The strain sensitivities of FBG1 to FBG5 range from 1.15 pm/με to 1.17 pm/με over a strain range of 0–924 με, with R^2^ values between 0.9997 and 0.9999. Detailed sensitivity and R^2^ results are shown in Table 1. These findings confirm that the push–pull multi-core optical fiber system effectively compensates for longitudinal strain.

### 3.3. Bending Sensing Characteristic

**Figure 8:**
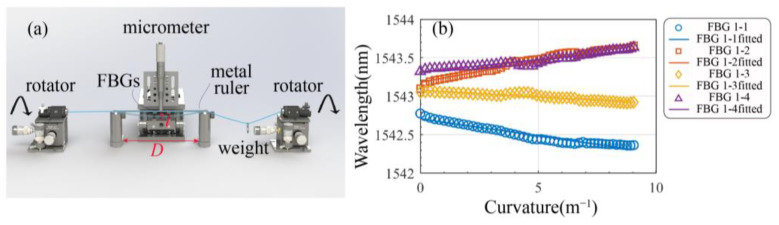
Characterization of enhanced sensitivity in bending sensing: (**a**) Schematic diagram of the experimental setup for bending measurement. (**b**) Relationship between the reflection wavelength and curvature for FBGs located at the same position.

A schematic of the vector bending-sensing experimental setup is shown in Figure 8a. The optical fiber is placed on the vector bending test platform, with both ends clamped and rotated using rotary fixtures. The FBG region is positioned beneath a spring steel strip of length D. Bending is induced by pushing downward on the strip using a precision micrometer screw gauge from above, enabling accurate curvature control. After each measurement, the rotary fixtures at both ends are rotated in 30° increments for multi-directional testing. The displacement l of the micrometer and the resulting bending curvature K follow the relationship

(11) $$\frac{1}{R}=\frac{8l}{D^{2}+4l^{2}}$$

Figure 8b presents the spectral responses of the push–pull bending sensing system at a bending direction of 0° under various curvature levels. It can be observed that within the same channel, the two FBGs exhibit redshift and blueshift, respectively, as the curvature increases from a straight to a highly bent state. This result is consistent with Equation (6).

## 4. Results and Discussion

**Figure 9:**
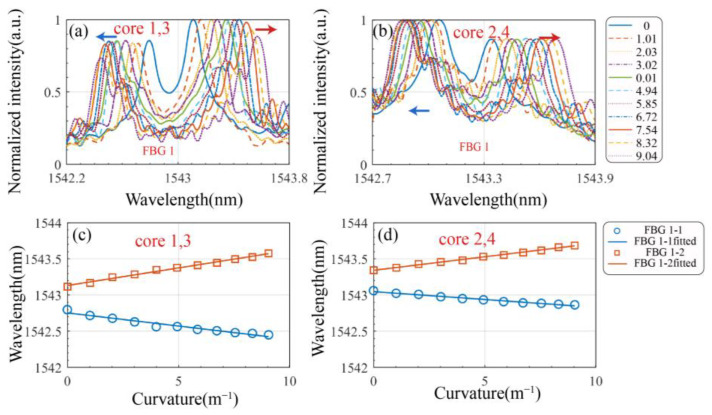
Enhanced bending sensitivity testing of the wearable glove: (**a**) Spectral response in channel 1. (**b**) Spectral response in channel 2. (**c**) Relationship between FBGs and curvature in channel 1. (**d**) Relationship between FBGs and curvature in channel 2.

We integrated the push–pull MCF sensor into a wearable glove to verify the enhancement in sensitivity. The spectral response of FBG1 on the glove is shown in Figure 9. Figure 9a,b display the redshift and blueshift in the FBG spectra in channels 1 and 2, respectively, as the bending curvature varies. The corresponding changes in Bragg wavelengths with bending curvature are illustrated in Figure 9c,d.

Taking cores 1 and 3 as an example, bending, strain, and temperature were decoupled based on Equations (3) and (4) and the calibration experiments, leading to the following relationship:

(12) $$\left\{\begin{array}{l} K_{\varepsilon 1}\approx K_{\varepsilon 2}\approx K_{\varepsilon 3}\approx K_{\varepsilon 4}=K_{\varepsilon }\\ K_{T1}\approx K_{T2}\approx K_{T3}\approx K_{T4}=K_{T}\\ \varepsilon_{r3}=-\varepsilon_{r1}\\ \Delta \lambda_{B1}=K_{\varepsilon }\varepsilon_{z}+K_{T}\Delta T+K_{\varepsilon }\varepsilon_{r1}\\ \Delta \lambda_{B3}=K_{\varepsilon }\varepsilon_{z}+K_{T}\Delta T+K_{\varepsilon }\varepsilon_{r3}\\ \Delta \lambda_{13}=\Delta \lambda_{B1}-\Delta \lambda_{B3}\approx K_{\varepsilon }\varepsilon_{r1}-K_{\varepsilon }\varepsilon_{r3}\approx 2K_{\varepsilon }\varepsilon_{r1} \end{array}\right.$$

From Equation (12), it can be observed that $\Delta \lambda_{13}$ is solely related to bending strain. By combining $\Delta \lambda_{13}$, $\Delta \lambda_{B1}$, and $\Delta \lambda_{B3}$, the axial strain $\varepsilon_{z}$, temperature change ΔT, and radial strain $\varepsilon_{r}$ can be directly solved. Subsequently, the bending vector can be calculated using Equation (6).

The bending sensitivity of the wearable glove is summarized in Table 2. In channel 1, composed of cores 1 and 3, the FBG bending sensitivities before decoupling strain and temperature effects were −36.319 pm/m^−1^ and 48.606 pm/m^−1^, respectively. After decoupling, the push–pull bending sensitivity reached 77.033 pm/m^−1^. In channel 2, formed by cores 2 and 4, the FBG bending sensitivities without decoupling were −21.729 pm/m^−1^ and 37.409 pm/m^−1^, respectively, while the decoupled push–pull sensitivity was 53.811 pm/m^−1^.

The decoupled push–pull sensitivities were derived from Equation (12). This demodulation method not only eliminates the influence of axial strain and temperature variation along the z-axis, but also significantly improves the sensitivity, accuracy, and stability compared to single-core demodulation—with sensitivity effectively doubled. The combined bending sensitivity based on channels 1 and 2 reached 93.967 pm/m^−1^.

**Figure 10:**
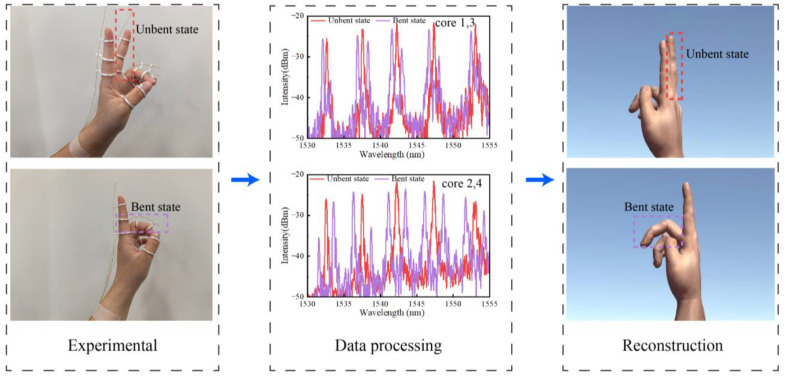
Gesture acquisition and reconstruction using the push–pull fiber-optic sensing-based wearable glove: FBG spectra and reconstructed gestures.

Figure 10 illustrates the process of single-finger gesture acquisition and reconstruction. A significant enhancement in sensitivity response can be clearly observed from the FBG reflection spectra. The gesture reconstruction was achieved by continuous shape estimation based on Equations (7) and (8), utilizing data from five sets of FBGs.

To better understand the advantages of our sensor compared to other types, we conducted a comparative analysis of capacitive, inductive, optical, and various FBG-based sensors. This comparison facilitates the evaluation of different technological solutions and helps identify the most suitable system for specific application scenarios. The resolution comparison highlights the real-world performance of each sensing technology, revealing their respective strengths and limitations. It is worth noting that most conventional sensors do not possess thermal decoupling capability. While this limitation has minimal impact on typical electrical sensors, it can introduce significant errors in temperature-sensitive FBG-based systems. In contrast, our sensor architecture incorporates an effective temperature–strain decoupling mechanism, which minimizes cross-sensitivity effects. This not only enhances the accuracy of strain and shape measurements under varying thermal conditions but also significantly improves the overall stability and reliability of gesture reconstruction. A detailed comparison of different sensor types is summarized in Table 3.

## 5. Conclusions

This study presents a push–pull enhanced wearable glove based on FCF, which was experimentally validated. The glove, based on shape-sensing principles, is user-friendly and highly portable. By fabricating a TRC at the fiber tip, symmetric cores were optically connected to enable push–pull sensing. This design significantly reduces the number of sensing channels required for data demodulation, allowing single-hand gesture sensing to be performed with a standard 16-channel interrogator. Furthermore, the bending sensitivity is substantially enhanced, enabling the detection of subtle gestures and motions. The decoupling of temperature and axial strain effectively minimizes the impact of environmental disturbances, improving accuracy and stability in gesture reconstruction. The compact and highly sensitive structure of the glove provides strong technical support for applications in precise motion analysis, rehabilitation monitoring, telemedicine, and remote diagnostics. Future research will focus on detecting combined wrist and finger movements and exploring the potential of wearable sensors for applications such as Parkinson’s disease tremor monitoring, rehabilitation devices, and human–computer interaction.

## Figures and Tables

**Table 1:** Strain sensitivity of FBGs.

FBGs	Channel 1		Channel 2	
	Sensitivity (pm/με)	R^2^	Sensitivity (pm/με)	R^2^
1-1	1.15	0.9999	1.16	0.9998
1-2	1.15	0.9998	1.16	0.9999
2-1	1.15	0.9999	1.16	0.9998
2-2	1.15	0.9999	1.16	0.9999
3-1	1.16	0.9999	1.16	0.9998
3-2	1.16	0.9998	1.16	0.9999
4-1	1.16	0.9998	1.17	0.9999
4-2	1.16	0.9998	1.17	0.9999
5-1	1.16	0.9997	1.17	0.9998
5-2	1.16	0.9998	1.17	0.9998

**Table 2:** Bending sensitivity of the wearable glove.

	FBG	FBG (pm/m^−1^)	Channel (pm/m^−1^)
**Channel 1**	Core 1-1	−36.319 48.606	77.033
	Core 3-1		
**Channel 2**	Core 2-1	−21.729	53.811
	Core 4-1	37.409	

**Table 3:** Comparison of different types of wearable glove sensors.

Sensor Type	Application	Sensitivity	Temperature Decoupling	System
Inductive [10]	Glove	Not mentioned	NO	Moderately
Capacitive [11]	Glove	Not mentioned	NO	Convenient
PDMS+PI+FBGs [23]	Arm	7.09 pm/°	NO	Moderately
PDMS+FBGs [25]	Wrist	24.90 pm/°	NO	Moderately
Photoelectric [26]	Glove	0.0125 nm/°	NO	Moderately
FBGs [27]	Forearm muscles	2.20938 pm/με	NO	Moderately
FBGs [28]	Forearm muscles	3.20794 pm/με	NO	Moderately
FCF-FBGs [This work]	Glove	93.967 pm/m^−1^	YES	Moderately

## Data Availability

Data are contained within the article.

## Abbreviations

The following abbreviations are used in this manuscript:

HCI	Human–computer interaction
FBGs	fiber Bragg gratings
MCF	multi-core fiber
FCF	Four-core fiber
FIFO	Fan-in/fan-out
MCD	Multi-channel demodulator
TRC	Tip-reflecting coupler
PDMS	polydimethylsiloxane
PI	polyimide
FBG	fiber Bragg grating
MCF-FBGs	multi-core fiber Bragg gratings
DCF	dual-core fiber
